# Are heavy metals in urban garden soils linked to vulnerable populations? A case study from Guelph, Canada

**DOI:** 10.1038/s41598-021-90368-3

**Published:** 2021-05-28

**Authors:** Fernando Montaño-López, Asim Biswas

**Affiliations:** School of Environmental Sciences, University of Guelph, Guelph, ON N1G 2W1 Canada

**Keywords:** Environmental impact, Geophysics, Environmental impact

## Abstract

With increasing population, there is growing concern for food security in urban areas. Though, urban gardening has gained popularity, several studies have found higher concentrations of contaminants in urban soils, especially heavy metals, often at toxic levels, which pose a potential risk for human health. Moreover, heavy metal polluted sites have been strongly associated with areas populated by low-income families, newcomers and racial minorities. In this study, heavy metals in the soils of community gardens in the city of Guelph, ON were examined as a case study and their relationship with vulnerable populations. We analyzed soil samples at two depths for a range of heavy metals and characterized their spatial patterns to see if they were related to disadvantaged communities. We estimated the pollution levels using two index-based approaches and assessed their potential risk for human health, although concentrations of most heavy metals were below the limits established by Canadian regulations, metals like Cd, Pb, Se and Zn exhibited a mild degree of pollution, whereas As exhibited a severe degree. Their association with vulnerable populations were weak, but hotspots were mainly located in low-income areas. This case study provides scientific evidence that could help to expand our understanding around the interconnection between pollution and poverty/racial inequality. Also the importance of generating strategies for the protection of human health and sustainable soil management practices in urban areas where food for human consumption is grown.

## Introduction

While UN projections place more than 65% of the global population in urban centers by 2050, healthy diets for many urban dwellers are constrained by limited incomes, rising food costs, and inequitable access to healthy and culturally appropriate foods; these are growing concerns in cities around the world^1^. Canadian cities are no exception^2^. Over the last few years, urban gardening has gained significant relevance due to its economic, social and cultural benefits; however, several studies in North America have shown that urban soils can have high concentrations of certain trace elements^3^. Since metals can enter the food chain from soil to groundwater or crops, urban soil contamination by heavy metals is of great concern in these areas as they may pose a potential risk for human health^4^.

High levels of contaminants in urban areas have been associated with site history and current land management practices^5^. Although there could be many sources that explain high heavy metal concentrations, some of the most common include: (1) deposition of small size particles, solid or liquid wastes; (2) incorporation of manufactured materials related to industry and (3) use of agrochemical inputs^6^. Soil test results and past factors can provide information to guide efforts to improve garden quality and protect the health of gardeners, their families, and other members in the community^5^.

Globally, there are more than 10 million sites of soil pollution reported, with > 50% of the sites contaminated with heavy metals and/or metalloids. It has been estimated that heavy metal pollution has a combined worldwide economic impact of more than US $10 billion per year^4^.Despite extensive soil heavy metal pollution reports in urban areas, these studies have mainly focused on their correlation with land use history^7–11^. In addition, several studies have shown the negative impact of contaminated sites on the health of vulnerable populations^12–16^. These environmental hazards, therefore, are not randomly distributed. Low-income populations^13^, newcomers^17,18^ and racial minorities^16,18^ tend to be more exposed to environmental issues with potential health effects.

Previous studies reported that some Toronto residents face environmental exposure to heavy metals, especially those that belong to vulnerable communities^17,19^. Langlois et al.^19^ studied Pb levels in Toronto children, the heavy metal exposure was explained by past industrial activities in the area. Guelph, located in southern Ontario in Canada, constituted along with Toronto and other centers, an area of tremendous industrial concentration during the middle of the twentieth century^20^.

Guelph is a medium-sized city with a population of about 132,000 inhabitants. With a history of settlement as early as the 1820s, the city has seen various uses of land over the years. In the past, Guelph was one of the most important manufacturing towns in South-Central Ontario with 48.8% of its population working for this sector by 1951, especially in iron, steel products, electrical apparatus and supplies^20^. Given background data on contamination due to land use across Southern Ontario, studying urban agricultural soils in Guelph was considered necessary. Making critical to develop a strategy to identify areas of high contamination and create alternatives to minimize the potential exposure of contaminants to disadvantaged neighborhoods.

Currently, there are a total of 28 community gardens used for raising vegetables. The soils of these gardens are rarely analyzed and yet, they could be contaminated with heavy metals. For example, McKeague et al.^21^ studied the level of minor elements in Canadian soils, finding Pb concentrations ranging from 12 to 71 ppm in sites near the city. While soil contaminants pose a risk to human health, the contaminated soils are often found in older and/or vulnerable neighborhoods located near the source of contaminants or which were previously zoned as industrial areas^18,22^.Little work has been done on mapping the spatial distribution of soil contaminants in urban agricultural settings and their association with vulnerable populations.. The present study was carried out on a regional scale to analyze the spatial pattern of heavy metal pollution in community gardens and better understand its relationship with disproportionate sociospatial bias that puts vulnerable populations in risk. Although there might not be a single explanation of these situations, these risks tend to cluster with one another, creating a long-term cumulative effect on environmental injustice and health disparities^23^.

## Materials and methods

### Study area and soil sampling

The study area, the city of Guelph, is in the province of Ontario, Canada. It is one of the fastest growing cities in Ontario with a very low unemployment rate and it attracts diverse populations. The urban area covers about 87 km^2^ with a population density of 1511 per km^2^. Guelph experiences cold winters and warm, humid summers with moderately high rainfall and snowfall. The city is situated on several drumlins of deglaciated landscapes.

**Figure 1:**
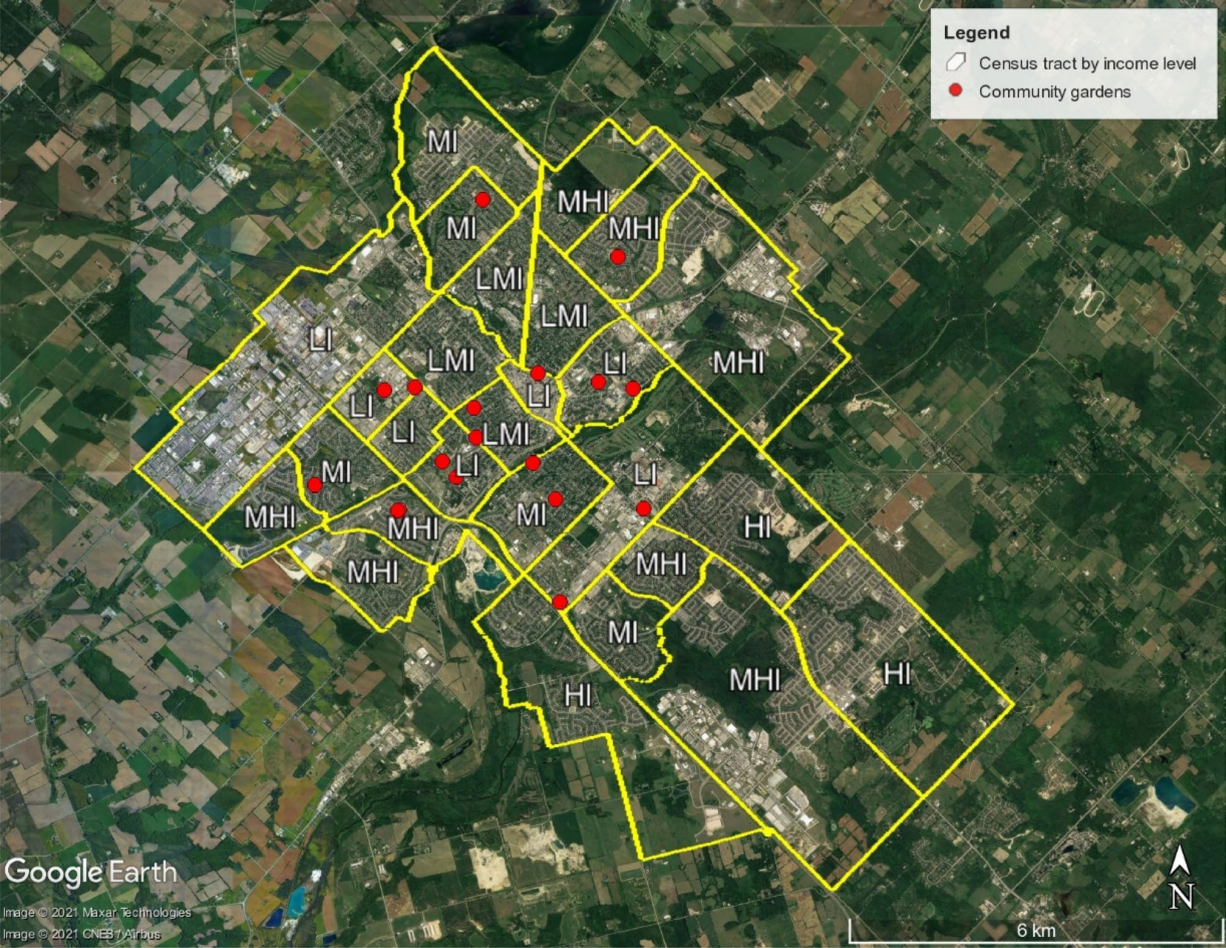
Map of the study area and sampling points. The map was prepared by using the “Places” tool in Google Earth Pro (https://www.google.com/earth/).

Due to its population diversity, to date there are 28 active community gardens in the city. In 2018, there were only 22 community gardens, which shows the increasing interest in growing food locally. A total of 40 surface (0–15 cm depth) and subsurface (15–30 cm depth) soil samples were collected with a hand auger from 20 participating community gardens (Fig. 1; two garden coordinators were inaccessible to collect soil samples) during the summer 2018. Each soil sample comprised a composite sample of seven subsamples taken across the community gardens. Samples were mixed thoroughly to make representative samples, sealed in polyethylene bags and taken to the laboratory for further analysis. In addition to the 40 composite samples, two soil cores of 5 cm tall and 5.7 cm diameter (volume—127.587 cm^3^) were collected from each community garden for moisture and bulk density analysis. Soil cores were collected from 5 to 10 and 20–25 cm depth representing the sampling depths.

### Physical and chemical analysis

Soil samples were air-dried, sieved through a 2-mm sieve and ground with a mortar and pestle to achieve a homogenous sample. Soil pH and EC were measured in distilled water, using a pH meter (Fisher Scientific, Model accumet AE150) and a digital conductivity cell (Fisher Scientific, Model accumet XL600), respectively, with a soil: solution ratio of 1:2. Soil bulk density was determined from previously collected undisturbed cores as mass per volume of oven dried soil. Soil texture was determined using the hydrometer method, separating very coarse sand, coarse sand, and medium sand with stacked sieves. As the content of organic matter was high in most of the samples, 25–30 g of soil were treated with hydrogen peroxide (30%) for OM removal, then left in Calgon solution (5%) overnight.

Organic matter was measured using the loss on ignition (LOI) method. Finely ground soil, which could pass through a 75 µm sieve was weighed (≅ 4 g) into porcelain boats. Samples were placed in a preheated 550 °C muffle furnace for 6 h. After 6 h, the muffle furnace was turned off and left to cool overnight. The next day, the porcelain boats were weighted, full and empty, and OM was calculated based on the weight difference of the sample.

Soil moisture was measured using the gravimetric method. Soil samples from two different depths (5–10 and 20–25 cm) were placed immediately in a polyethylene sealed bag. The sample was weighed as received in the laboratory and again after drying in an oven for 24 h at 105 °C. Heavy metal concentration was measured in the Soil and Water Testing Laboratory at McGill University. Previously processed and finely ground 0.160 g of soil sample was mixed with 2 mL of HNO_3_ (70%) and let stand overnight. Then, on dry block, samples were slowly heated to 120 °C for 5 h. Once cooled to room temperature, samples were transferred to polypropylene tubes for a final volume of 50 mL using nanopure water and mixed thoroughly. Next day, samples were diluted to 20 × and HNO_3_ was added to a final 1% v/v nitric acid concentration. Total concentration was measured on a Varian ICP-MS model 820 MS (Analytik-Jena, Germany). For quality assurance and quality control (found in Supplementary Material), blanks were run every 12 samples to avoid background noise impact, parallel replication of samples were simultaneously determined, and standard reference materials were provided by Environment Canada, a national regulatory body.

### Statistical data and analysis

We collected sociodemographic data from the 2016 Census done by Statistics Canada. This data provided information about median total income of households, percentage of immigrant population and percentage of visible minorities in a census tract level. All data analyses were performed using Microsoft Office Excel 2016. GIS-based approach was used to characterize the spatial distribution of heavy metals at the regional scale. The spatial distribution using inverse distance weighted (IDW) was performed using ESRI ArcGIS 10.4. To associate heavy metal pollution with sociodemographic data, we carried out Pearson’s product-moment correlation. For this, we estimated the centroid from the spatial distribution maps of metals with mild to severe degree of pollution to obtain a representative value for every census tract area. These areas are divided in low (LI), low to middle (LMI), middle (MI), middle to high (MHI) and high income (HI).

## Results and discussion

### Physico-chemical parameters

The soil properties, including pH, EC, texture, and organic matter (OM) content varied from garden to garden and across depths. Values of pH ranged from 7.31 to 8.03 with a mean value of 7.73. These suggested neutral conditions tended to vary to slightly alkaline conditions for all soil samples. Similar pH characteristics (near neutral, 6.5–7.5) in urban gardens and the unavailability of many metals for ready uptake into plant tissues under these conditions have been described by the Cornell Waste Management Institute^24^. This is probably the most important factor controlling the uptake of heavy metals^25^. Soil EC exhibited a large range. The minimum value was 366.322 μS cm^−1^ and the maximum was 2713.69 μS cm^−1^, with the mean value of 822.11 μS cm^−1^, classifying most samples as nonsaline according to Boulding^26^.

For particle size distribution, sand size fraction was the highest, followed by silt and clay. Guelph soils have been described as predominantly loams with some small areas of sandy loam. The higher sand content in these soils may be due to the influence of the outwash sands which surround them^27^.

The OM content ranged from 5.62 to 22% with an average value 11.22%. According to Impellitteri et al.^28^, the complex interactions between heavy metals and soil organic matter result in changes in solubility, mobility and bioavailability of these elements. Generally, the solid phase organic matter is associated with retention, decreased mobility and reduced bioavailability of trace metals.

Bulk density and moisture were determined for the soil samples collected from 5 to 10 and 20–25 cm depth. Bulk density values fluctuated between 0.644 and 1.14 g cm^−3^. Moisture ranged from 8.13 to 48.34% with a mean value of 22.91%.

### Sociodemographic of Guelph and community gardens

An initial assessment of sociodemographic information made available by Statistics Canada (2017,^29^), shows that Guelph is a city with around 132,000 inhabitants in which the average household size is 2.5 people. The median total income of households is 77,984 CAD, an amount below the national median (81,347 CAD). The total visible minority population is around 24,500 people, defined as “persons, other than Aboriginal peoples, who are non-Caucasian in race or non-white in colour”. And the immigrant population is around 28,000 people, this category is defined as “persons who are, or who have ever been, landed immigrants or permanent residents”.

At the census tract level, the median household income ranges from 45,517 to 112,085 CAD. When this range is divided in five proportional categories of income, 50% of the gardens are located in areas with the lowest income. This proportion could indicate the importance of urban agricultural production for low-income neighborhoods. In terms of immigrant population cultivating food in the city, data shows the top two categorized areas inhabited with more immigrant population hold 27% of gardens, however the categories with less immigrant population hold 66% of the community gardens. Regarding the proportion of visible minorities, the areas with less percentage of visible minorities have 55% of the total gardens, while the areas with more visible minorities have 38% of the gardens.

### Heavy metals in community gardens

The descriptive statistics of metal concentrations at 0–15 and 15–30 cm depths are presented in Tables 1 and 2, respectively. Concentration of heavy metals varied among the community gardens. Mean concentrations of Co, Ni, Cu, Ba and Cr were below the limits for garden soils according to the Canadian Council of Ministers of the Environment (CCME^30^) soil quality guideline for agricultural land use that were established to protect agricultural production and to maintain human health. However, some sites showed high levels of contamination for certain elements. For Zn, 17.5% of the samples exceeded the CCME limits. Similarly, for Pb, 15% of the sites sampled showed concentrations above the limits for food production established by the CCME, while for As, two samples showed higher concentrations than permissible limits and for Cd, only one sample surpassed the limits. However, for Se, 45% of the study sites showed concentrations above 1 ppm. A wide range of concentration of these metals could point to an anthropogenic source of contamination.

**Table 1:** Descriptive statistics of the soil heavy metal concentrations at 0–15 cm in Guelph community gardens (in ppm).

Variable	Zn	Rb	Sr	Cd	Ce	Pb	As	Se
Mean	196.04	13.00	52.85	0.74	32.66	43.66	4.68	0.92
SD	138.26	2.22	23.92	0.35	4.79	38.10	10.39	0.85
Median	132.21	13.06	51.79	0.62	31.48	28.84	2.22	0.64
Minimum	96.58	8.81	18.17	0.38	22.97	15.74	ND*	ND*
Maximum	502.91	17.44	96.00	1.84	41.22	151.02	47.41	3.00
CV	70.53	17.06	45.27	46.81	14.67	87.28	222.21	92.24
CCME limits	250	–	–	1.4	–	70	12	1

*ND non-detectable.

**Table 2:** Descriptive statistics of the soil heavy metal concentrations at 15–30 cm in Guelph community gardens (in ppm).

Variable	Zn	Rb	Sr	Cd	Ce	Pb	As	Se
Mean	184.85	12.90	55.56	0.71	34.75	38.56	4.22	0.97
SD	144.20	2.19	29.33	0.26	6.84	30.01	7.15	0.82
Median	124.96	13.05	47.28	0.63	34.19	26.02	2.82	0.64
Minimum	81.59	8.82	16.83	0.38	23.08	14.47	ND*	ND*
Maximum	546.67	16.74	136.04	1.23	52.50	124.41	31.57	3.11
CV	78.01	16.97	52.78	35.93	19.68	77.82	169.71	84.70
CCME limits	250	–	–	1.4	–	70	12	1

*ND non-detectable.

Different concentrations of minor elements were also found in the studied community gardens including Cerium, Strontium and Rubidium. No information about CCME limits is available for these metals; this may be due to their unknown influence. According to Ramos et al.^31^, current information is not enough to determine the safe levels of exposure in humans. However, higher concentrations of these elements in this study pose a question for assessment of these metal`s influence and inclusion in the CCME guideline. Data on background levels is essential for assessment of the degree of soil contamination as the elements can be part of the nutritional chain form soil to plants to animals, including humans.

### Potential public health risks in vulnerable populations

#### Zinc

Concentrations of Zn in community gardens in Guelph variated from 81.59 to 546.67 ppm at two depths, while the limit established by the CCME is 250 ppm. High concentrations of Zn were found in the central downtown area of the city. This region is characterized by past intense industrial activity and may contribute to the high heavy metal concentration of the soils. Besides the anthropogenic contributions, mineral deposits of sphalerite (a Zn rich mineral) have been found across the central part of Guelph^32^.

**Figure 2:**
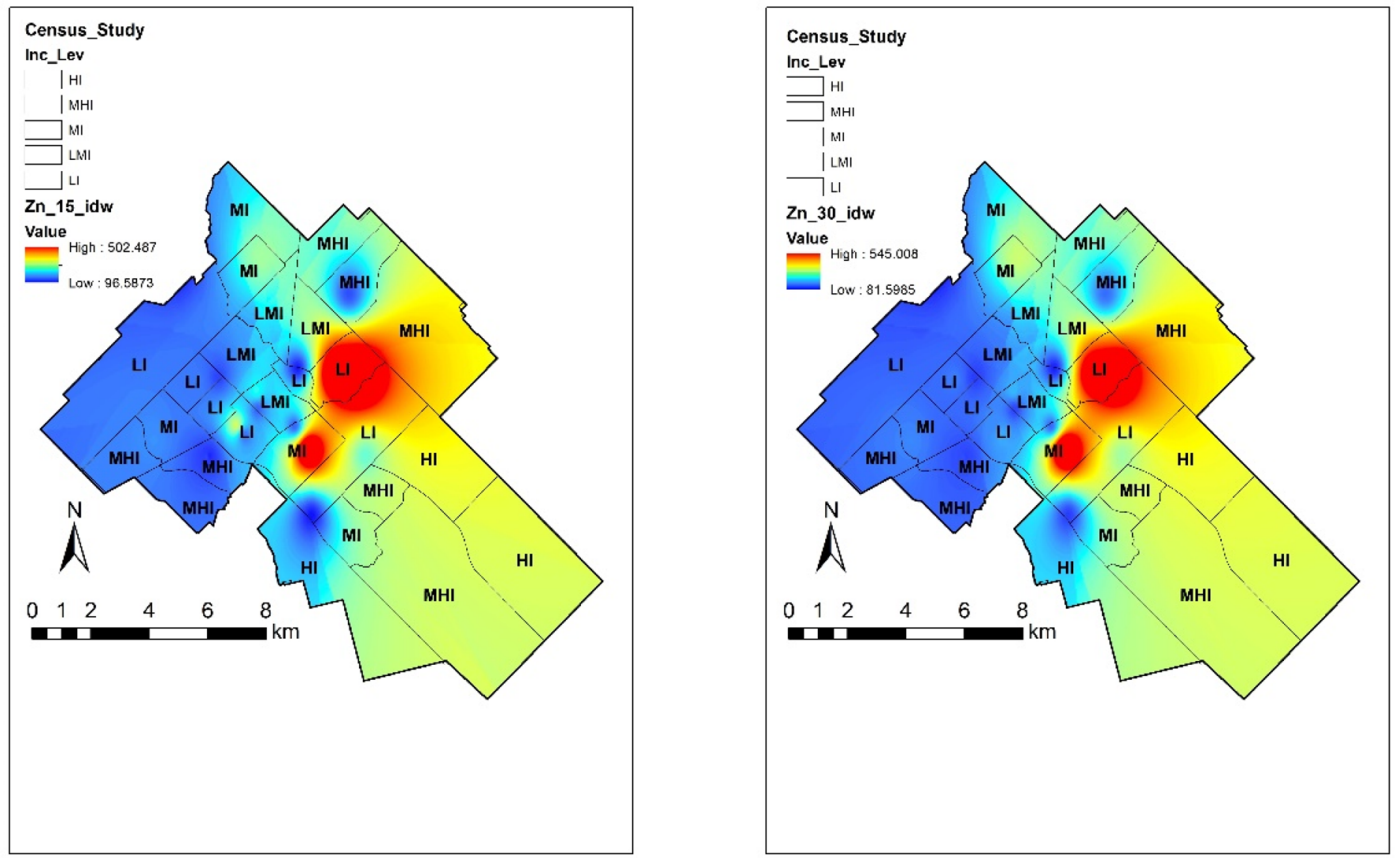
Spatial distribution of Zn at 0–15 and 15–30 cm depth in urban gardens of Guelph in relation with income level. The map was prepared by using the “Geostatistical Analyst” tool in ArcGIS 10.4 (https://desktop.arcgis.com/en/).

The behaviour of this metal in surface soils in relationship with the sociodemographic data shows that it is weakly associated with low income (r = 0.12), and a weak negative association with both percentage of immigrants (r = − 0.32) and percentage of minorities (r = − 0.28). In subsurface soils the correlations with low-income neighborhoods (r = 0.16), immigrant (r = − 0.32) and minority population (r = − 0.27) behaved similarly. The spatial distribution of this element by income level of census tract areas is found in Fig. 2.

According to Alloway^6^, Zn is naturally present in all soils with typical background concentrations of 10–100 mg kg^−1^ or ppm. Human activities have enriched top soils and Zn toxic soils are less widespread than deficient ones. Zn contaminated soils are unlikely to pose a risk to humans as the phytotoxic effects may limit transfer of excessive Zn to the human food chain. Therefore, risk assessment of Zn contaminated soils focuses on its ecotoxicological effects on soil organisms. However, the long-term exposure for residents in areas with Zn concentrations above the CCME limits Zn can experience negative impact on organs like the brain, causing lethargy and focal neuronal deficits; in the respiratory tract, it can cause respiratory disorder after inhalation of Zn smoke and metal fume fever; and in the gastrointestinal tract, it may cause nausea/vomiting, epigastric pain and diarrhea. It can also pose an elevated risk of prostate cancer^33^.

#### Cadmium

**Figure 3:**
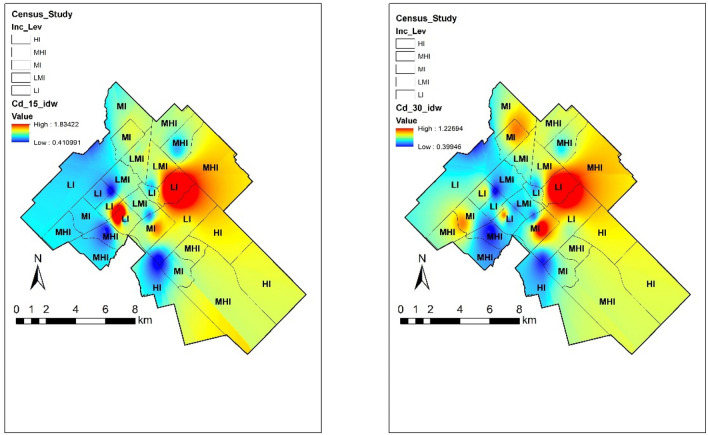
Cd content in 0–15 and 15–30 cm depth with varying income levels. The map was prepared by using the “Geostatistical Analyst” tool in ArcGIS 10.4 (https://desktop.arcgis.com/en/).

Cd is a non-essential metal that is naturally present in all soils as a divalent cation at concentrations typically ranging between 0.1 and 1.0 ppm. The presence of Cd is unlikely to affect the chemistry of soil^6^. However, given its pronounced toxicity, Cd can affect ecosystem functions at trace levels. Additionally, the toxicity of Cd in soil is persistent, since its residence time exceeds decades and its bioavailability does not decrease over time^34^. In this study, the concentration of Cd was low compared to the limits established by the CCME except for one garden with a concentration of 1.4 ppm. However, monitoring of this metal is essential for future management practices. Figure 3 illustrates the spatial distribution of Cd in Guelph. Cd hotspots were detected in low-income areas of the downtown area of Guelph, in which higher levels of Cd may be ascribed to anthropogenic activities. Previous studies have shown that Cd concentrations in soil are closely related to foundry and smelting activities^35,36^, these types of industries played an important role in sites near the affected zone. Pearson correlation coefficients showed that Cd is not correlated with income levels in surface (r = − 0.009) and subsurface (0.035) soils. While also showing weak negative correlations with immigrant (r = − 0.39 and r = − 0.23) and visible minority populations (r = − 0.35 and r = − 0.20) in both depths.

However, in the area with high Cd concentration, low-income dwellers can face negative health effects associated with Cd exposure. According to the ATSDR^37^, eating food or drinking water with very high Cd levels severely irritates the stomach, leading to vomiting and diarrhoea, and sometimes even death. Eating lower levels of Cd over a long period of time can lead to a build-up of Cd in the kidneys and exposure to low levels of Cd over a long time can also cause bones to become fragile and break easily. It has also been determined that Cd and its compounds are human carcinogens.

#### Lead

**Figure 4:**
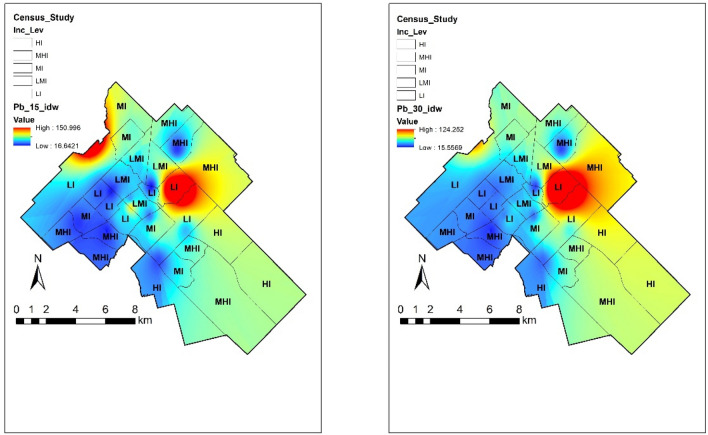
Concentration of Pb around the city in relation with income. The map was prepared by using the “Geostatistical Analyst” tool in ArcGIS 10.4 (https://desktop.arcgis.com/en/).

According to Alloway^6^, Pb exists predominantly in the + 2 oxidation state and it may remain bioavailable for a long period of time. The chemical behaviour of Pb in soil depends very much on the organic matter content. Pb is strongly adsorbed on organic matter at pH 5 and above^24^. Although most of the community gardens showed relatively low concentrations of Pb, around 15% exceeded the limits established in Canada with the highest concentration of 151.02 ppm at the top 15 cm soil. Figure 4 shows different concentrations of Pb across Guelph by income level in two different depths. The most heavily polluted sites found in the northeast and downtown part of the study area were associated with a site history that includes important industrial activity, mineral deposits of galena (a Pb mineral) and over a century of agricultural activities which could be associated with the use of agrochemicals containing Pb.

Similar to previous metals mentioned, correlation coefficients showed very weak association with low income (r = 0.03 and r = 0.04) and negative weak link with immigrant (r = − 0.39 and r = − 0.35) and visible minority (r = − 0.37 and r = − 0.31) populations at both depths sampled. Although there was a very low association with vulnerable populations, an important hotspot is located within a low-income neighbourhood. Special attention should be paid in that zone to small children due to their vulnerability to Pb toxic consequences. These consequences may cause negative lifelong effects on their nervous system. Also, long-term exposure of Pb for adults, produces risk of high blood pressure and kidney damage. Finally, complications during pregnancy can involve the mother’s and baby’s health^38^.

#### Arsenic

**Figure 5:**
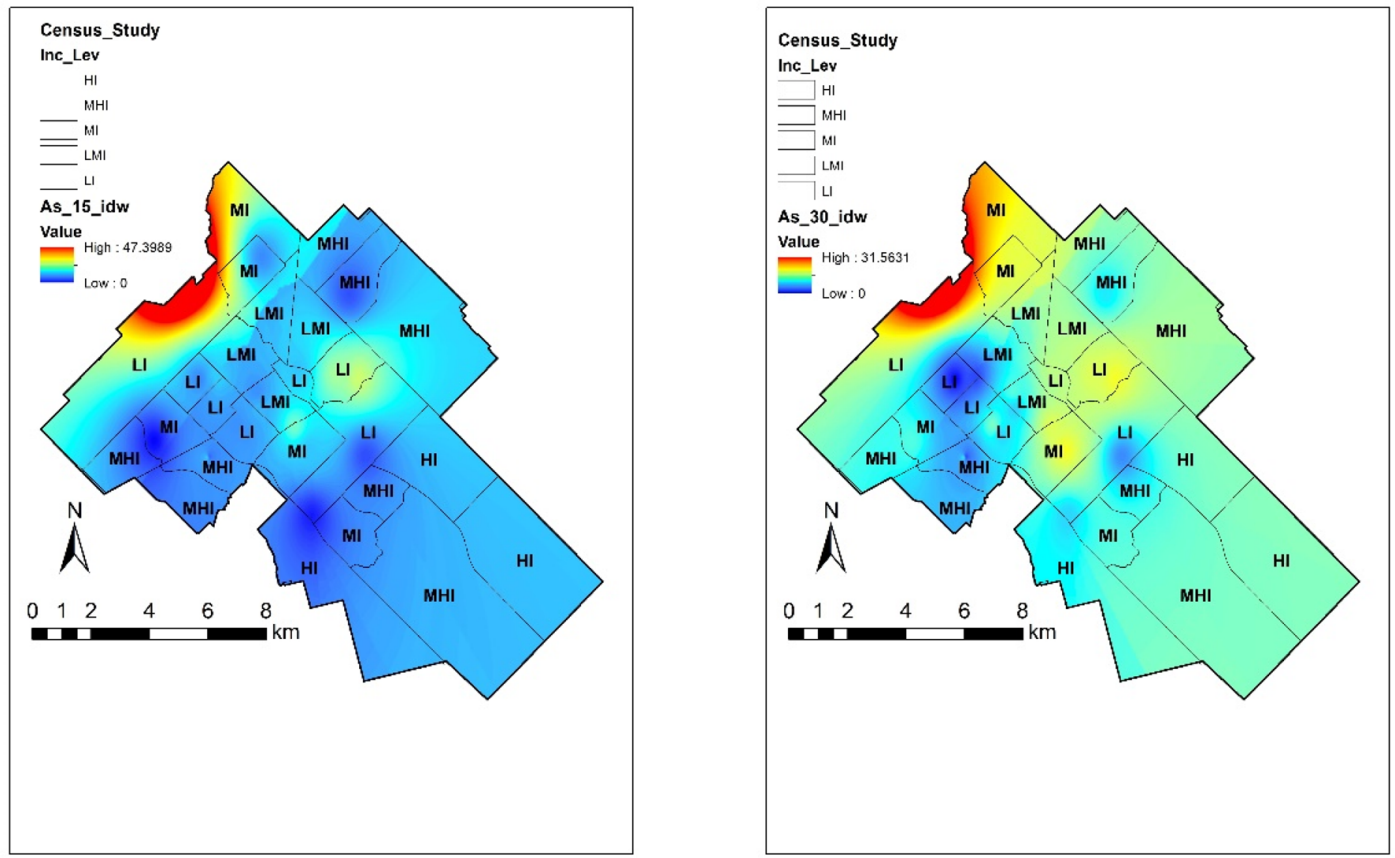
As concentrations across Guelph in two different depths and its association with low-income communities. The map was prepared by using the “Geostatistical Analyst” tool in ArcGIS 10.4 (https://desktop.arcgis.com/en/).

As is ubiquitous in nature, occurring in most soils and rocks at detectable quantities. As exists in more than one oxidation state; and bonds with sulphur, organic matter and carbon more readily, and undergoes biological transformations resulting in volatilization from soil^6,39^. There was only one garden that surpassed 12 ppm of permissible limit set by CCME. Moreover, these concentrations were 31.57 and 47.41 ppm, much higher than the set limit indicating a possible toxic area within the city. Figure 5 shows the spatial distribution of As in Guelph. The elevated concentration in the northeast part of the region could have been caused by the past use of agrochemicals; however, this elucidation requires further investigation.

As distribution in relation with vulnerable populations showed negative and weak correlation for all sociodemographic variables. Pearson correlation values show that there is not a strong relationship with low income (r = − 0.26, r = − 0.05), immigrant communities (r = − 0.31 and r = − 0.38) and visible minorities (r = − 0.38 and r = − 0.43) in the two different depths of this study. Even though only one site was heavily polluted with As, constant monitoring of this should be in place. The potential risks associated for residents of that area include the decrease in production of white and red blood cells. This can lead to damages in the circulatory and nervous system. Skin associated problems (like redness and swelling) may occur when direct dermal contact with As is produced. It is well-known that inorganic As is carcinogenic to humans, while organic As compounds show lower toxicity to humans as compared to its inorganic form^40^.

#### Selenium

**Figure 6:**
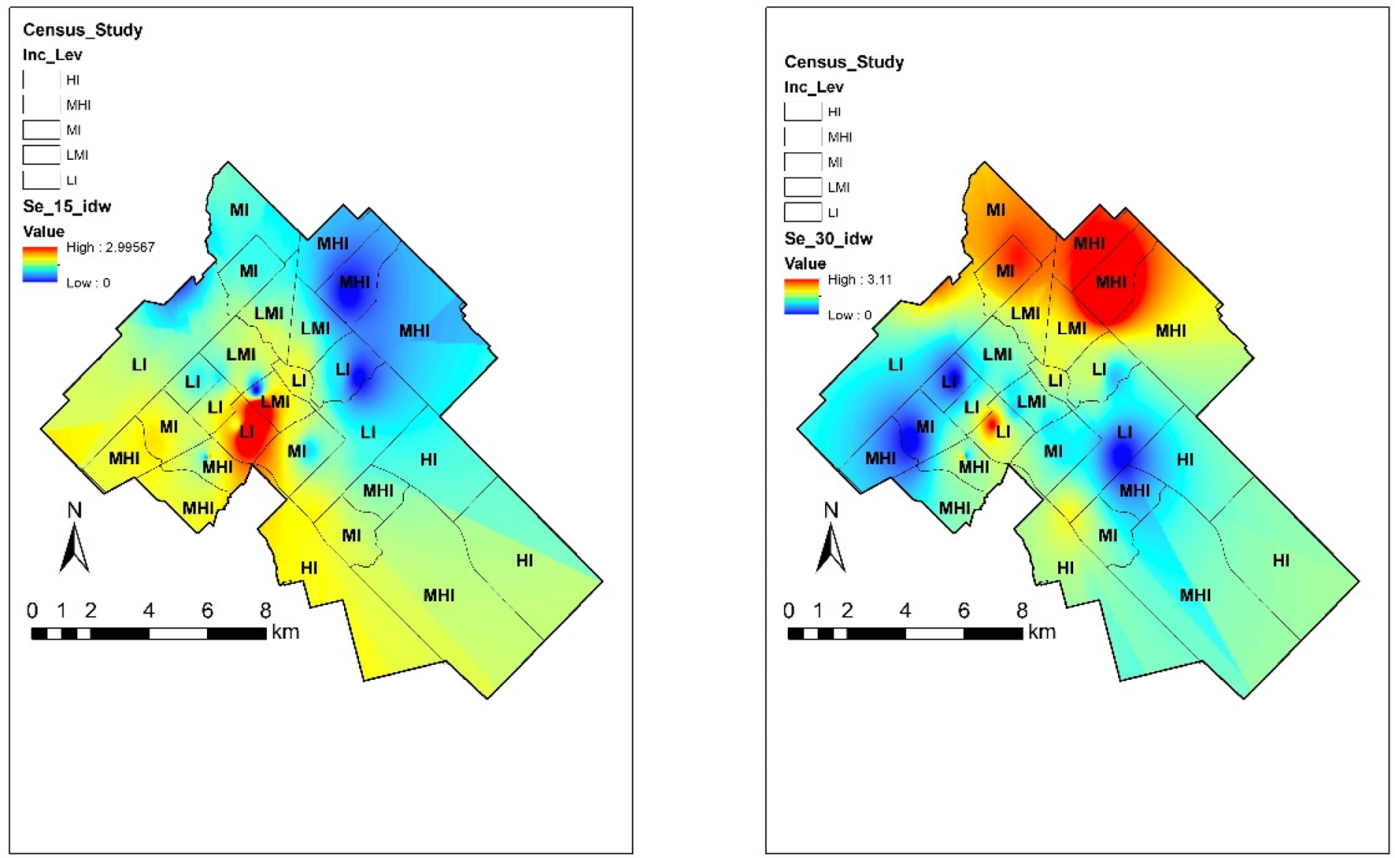
Spatial distribution of Se in Guelph in the 0–15 and 15–30 cm depth with varying income conditions. The map was prepared by using the “Geostatistical Analyst” tool in ArcGIS 10.4 (https://desktop.arcgis.com/en/).

Se in soil is found in different forms and oxidation states. Typical levels of Se in soil range from 0.01 to 2 ppm. However, due to Se partitioning on its solid phase, concentration values do not portray the effects of this element on the environment. Increasing amounts of Se in the environment have been recorded and attributed to human and natural activities^41^. In almost half of the samples, Se showed higher concentrations than the permissible limit of CCME, showing a more threatening distribution in Guelph. Figure 6 shows these concentrations in the city in association with low-income areas. Pearson correlation coefficients show different patterns in both depths. In surface soils, low income had a negative weak association (r = − 0.21) while immigrant (r = 0.06) and minorities (r = 0.06) had very weak relation. For subsurface soils, low-income had a positive weak correlation and immigrant communities (r = − 0.42) and visible minorities (r = − 0.43) in the area showed a weak negative correlation. The spatial distribution of Se presented different patterns, high levels in surface soil were found in the central area while hotspots in subsurface soil were found in the northeast of Guelph. These areas are linked to previous industrial activities such as coal combustion, oil processing and smelting, known as anthropogenic sources of Se^41^.

Se behaviour in the environment is mainly dependent on its concentration level and speciation. The latter, mostly controlled by redox potential and pH which regulates its mobility in soil^41^. Attention should be paid in those areas with concentrations above the CCME limits, which could face “selenosis”, a condition that results from high Se level in blood, its symptoms include irritability, hair loss, dermal and neurological damage^42^.

### Assessment of pollution in community gardens

The single pollution index can be used to evaluate the degree of heavy metal pollution in soil. This method measures pollution by a single element and refers to the ratio between the obtained value of the target element and the standard limit value of that element, in this case the limits established by the CCME. The higher the index value, the higher the degree of pollution. It is estimated using Eq. (1):

1 $$Pi = \frac{Ci}{{Si}}$$

where Pi is the environmental pollution index of pollutant i for the soil, Ci is the concentration of soil heavy metal i and Si is the pollution limit. The statistical results of the Pi value for each heavy metal (both depths considered) are represented in Table 4 where only those heavy metals that exceeded permissible limits were evaluated. The single pollution index grading standards are provided in Table 3^43^.

**Table 3:** Single pollution index grading standards (adapted from Wang et al.^43^).

Level	Single pollution index (Pi)	Degree of pollution
I	Pi ≤ 1	Non pollution
II	1 < Pi ≤ 2	Mild pollution
III	2 < Pi ≤ 3	Moderately polluted
IV	Pi > 3	Severe pollution

The average Pi values for Zn, Pb, Cd and Se were less than two but more than one, revealing mild contamination. The mean Pi value for As was 3.291, indicating possible severe pollution in the study area.

Another index is the Nemerow Pollution Index (NPI), which gives us more information about the overall degree of contamination of the soil and includes the contents of all analysed heavy metals ^44,45^. The NPI can be calculated using Eq. (2) as:

2 $$PI_{Nemerow} = \sqrt {\frac{{\left( {\frac{1}{n}\mathop \sum \nolimits_{i - 1}^{n} PI} \right)^{2} + PI_{max}^{2} }}{n}}$$

where PI is the calculated values for the Single Pollution Index, PI_max_ is the maximum value for the Single Pollution Index of all heavy metals and *n* the number of heavy metals. Table 4 shows the value calculated for all the community gardens in Guelph in which certain elements exceeded the limits established for agricultural production in Canada. This index is a good indicator of total contamination because it highlights the most contaminated elements, is widely used, considers all individual elements, and has a precise scale. Table 5 presents five classes of soil quality based on the NPI^44^.

**Table 4:** Statistical analysis of the single pollution index (Pi) of each heavy metal surpassing the limits.

Element	Pi			
	Minimum	Maximum	Average	Pollution degree (average)
Zn	1.023	2.187	1.866	Mild
Pb	1.064	2.157	1.635	Mild
As	2.631	3.951	3.291	Severe
Cd^a^	1.312	1.312	1.312	Mild
Se	1.059	3.106	1.671	Mild
Nemerow Pollution Index	1.196			

^a^Only one sample exceeded the CCME limits for this element.

**Table 5:** Nemerow Pollution Index soil classes (adapted from Kowalska et al.^44^).

Class	NPI value	Quality of soil
I	< 0.7	Clean
II	0.7–1	Warning limit
III	1–2	Slight pollution
IV	2–3	Moderate pollution
V	> 3	Heavy pollution

As shown at the bottom of Table 4, the Nemerow Index classifies contaminated soils in the community gardens as slightly polluted. Further study is required in order to understand completely those sites where the concentrations were higher and to determine the processes and possible long-term impacts on the nearby population.

## Conclusion

In this study, we characterized heavy metals in community gardens from Guelph and investigated the potential health risks for vulnerable communities. Pollution evaluation and correlation with sociodemographic data were also carried out. The results showed that most average concentrations of elements analysed in garden soils of the city are lower than the Canadian soil quality guidelines for the protection of the environment and human health. However, Zn, Pb, Se, Cd and As were found to be at higher concentrations than usual. In this region, the single pollution index of heavy metals showed that Zn, Pb, Se and Cd represent mild contamination, whilst As has a severe pollution degree. The Nemerow pollution index analysis which allows the assessment of the overall degree of pollution, suggested that community gardens in Guelph present slight contamination.

The high concentrations of heavy metals in some parts of the city are attributed mainly to past industrial activities, which have been documented previously. We have also characterized the spatial distribution of the patterns of heavy metals with GIS-based mapping tools which proved to be useful in improving the interpretation of the statistical output in tandem with sociodemographic information. Although many heavy metals were weakly associated with vulnerable populations, hotspots were mainly located in low-income areas of the city. Data provided shows that those hotspots tend to occur more in areas with low earning households, alternatively the same hotspots were not mainly located in zones with high number of immigrants and visible minorities. Similar results were obtained by Premji et al.^46^, their pollution measures were negatively correlated to income and no association was seen with visible minority and immigrant population. This shows diverse patterns of poverty and race, which could have several implications and may be the reason why little work has been done in the country on the relationship between contamination and vulnerable neighbourhoods.

Finally, special attention should be given to the constant monitoring of heavy metals and their relationship with vulnerable populations. Further local studies examining plant material and the pathway of these elements into human body are advised. While there remains limitations in this study, the outcomes provide critical data to better study the interconnection between pollution in urban agricultural areas and low income, racial inequality and many other factors that promote environmental injustice. Importance should also be given to the formulation of policies aiming to: (1) reduce the potential harm of heavy metals to humans and (2) constant monitoring of urban agricultural areas that might not be connected to community farming (such as privately-owned gardens where food is grown).

## Supplementary Information

Supplementary Information.
