# Supplementary material for: Are heavy metals in urban garden soils linked to vulnerable populations? A case study from Guelph, Canada
Source: Sci Rep. 2021 May 28;11:11286. doi: 10.1038/s41598-021-90368-3 (PMC8163869; doi:10.1038/s41598-021-90368-3)
Supplement: Supplementary file 1 — Supplementary Information. [file 41598_2021_90368_MOESM1_ESM.docx]

**Heavy metals in urban garden soils pose potential health issues in historically industrialized cities**

Fernando Montaño-López and Asim Biswas

Supplemental Material

| Quality control report: | | | | | | | | | | | | | | | | | | | | | | |
| --- | --- | --- | --- | --- | --- | --- | --- | --- | --- | --- | --- | --- | --- | --- | --- | --- | --- | --- | --- | --- | --- | --- |
| *External control samples from Environment Canada (see reference below)* | | | | | | | | | | | | | | | | | | | | | | |
|  |  |  | **Na** | **Mg** | **Al** | **K** | **Ca** | **Mn** | **Co** | **Ni** | **Cu** | **Zn** | **Rb** | **Sr** | **Cd** | **Ba** | **Ce** | **Pb** | **Cr** | **Fe** | **As** | **Se** |
| 180815075 | Biswas QC 1 | 90.3 | 259 | 11086 | 17444 | 3734 | 41470 | 1099 | 13.83 | 54.20 | 80.10 | 1407 | 27.71 | 77.42 | 4.41 | 151.28 | 35.95 | 228.63 | 83.77 | 49545 | 12.46 | 2.33 |
| 180815036 | Biswas QC 1 | 90.3 | 192 | 10806 | 16707 | 3539 | 39261 | 991 | 12.03 | 47.03 | 67.53 | 1207 | 25.65 | 72.14 | 4.33 | 134.20 | 31.57 | 272.29 | 92.13 | 47461 | 13.14 | 6.53 |
|  | ref value |  | 204 | 11900 | 18500 | 3600 | 42600 | 1070 | 12.30 | 48.00 | 72.90 | 1310 |  | 73.60 | 3.70 | 131.00 |  | 222.00 | 81.00 | 54400 | 15.30 | 1.29 |
|  |  |  |  |  |  |  |  |  |  |  |  |  |  |  |  |  |  |  |  |  |  |  |
| 180815076 | Biswas QC 2 | 92.1 | 308 | 10720 | 25251 | 5653 | 23225 | 1384 | 16.86 | 68.77 | 84.09 | 1776 | 42.44 | 53.19 | 3.03 | 217.80 | 45.59 | 97.30 | 83.68 | 40398 | 14.64 | 1.35 |
| 180815114 | Biswas QC 3 | 92.1 | 307 | 10128 | 25871 | 5703 | 22231 | 1371 | 16.83 | 65.20 | 79.80 | 1743 | 42.31 | 52.57 | 2.99 | 231.10 | 43.23 | 65.16 | 84.73 | 37933 | 15.95 | 1.59 |
| 180815154 | Biswas QC 4 | 92.1 | 393 | 9839 | 22638 | 6091 | 22919 | 1415 | 18.39 | 72.33 | 93.41 | 1834 | 39.17 | 49.07 | 2.95 | 205.61 | 43.04 | 107.81 | 72.51 | 38233 | 19.74 | 4.85 |
|  | ref value |  | 255 | 11500 | 24100 | 4610 | 23600 | 1430 | 15.40 | 64.80 | 78.80 | 1818 |  | 44.70 | 2.41 | 188.00 |  | 98.50 | 70.00 | 39200 | 17.30 | 1.20 |
|  |  |  |  |  |  |  |  |  |  |  |  |  |  |  |  |  |  |  |  |  |  |  |
| 180815115 | Biswas QC 5 | 98.2 | 544 | 9904 | 19354 | 5144 | 59733 | 2145 | 13.90 | 46.29 | 88.28 | 1419.96 | 32.96 | 135.65 | 4.32 | 164.55 | 36.82 | 108.99 | 103.15 | 45338 | 4.16 | 3.88 |
|  | ref value |  | 536 | 11050 | 18200 | 5260 | 59950 | 2248 | 12.00 | 45.00 | 83.10 | 1370 |  | 120.00 | 3.53 | 138.00 |  | 168.00 | 102.00 | 48000 | 13.80 | 4.12 |

| External control samples are issued from: | | |  |
| --- | --- | --- | --- |
| Environment Canada - Proficiency Testing Program | | | |
| Trace Elements in Sediments Studies | | |  |
| Information and Quality Management | | |  |
| Water Science and Technology Directorate | | |  |
| Burlington, ON, CANADA | |  |  |
| [Ptstudies@ec.gc.ca](mailto:Ptstudies@ec.gc.ca) |  |  |  |

| *Method Replication of digestions* | | | | | | | | | | | | | | | | | | | | | | | |
| --- | --- | --- | --- | --- | --- | --- | --- | --- | --- | --- | --- | --- | --- | --- | --- | --- | --- | --- | --- | --- | --- | --- | --- |
|  |  | Sample Label | client ID | Na | Mg | Al | K | Ca | Mn | Co | Ni | Cu | Zn | Rb | Sr | Cd | Ba | Ce | Pb | Cr | Fe | As | Se |
| 180815048 |  | Biswas 2 | GP | 213 | 24202 | 10503 | 2320 | 64491 | 481 | 5.08 | 15.50 | 28.51 | 135.52 | 13.68 | 76.93 | 0.93 | 61.95 | 32.36 | 25.60 | 22.87 | 14537 | 3.48 | 1.94 |
| 180815104 |  | Biswas 41 | GP | 196 | 14412 | 10506 | 2145 | 48529 | 565 | 6.02 | 14.80 | 40.83 | 172.89 | 12.92 | 75.18 | 1.17 | 70.91 | 31.25 | 26.74 | 29.63 | 14346 | 1.17 | ND* |
|  |  |  |  |  |  |  |  |  |  |  |  |  |  |  |  |  |  |  |  |  |  |  |  |
| 180815052 |  | Biswas 6 | GG | 782 | 13281 | 14200 | 1634 | 39452 | 530 | 5.35 | 13.17 | 24.64 | 88.78 | 16.37 | 90.95 | 0.61 | 62.17 | 52.50 | 17.16 | 16.32 | 16851 | 5.47 | 0.56 |
| 180815105 |  | Biswas 42 | GG | 330 | 15585 | 11437 | 1434 | 39138 | 545 | 5.69 | 12.83 | 20.16 | 95.19 | 14.13 | 39.64 | 0.55 | 61.15 | 36.19 | 20.15 | 19.70 | 16415 | 5.67 | ND* |
|  |  |  |  |  |  |  |  |  |  |  |  |  |  |  |  |  |  |  |  |  |  |  |  |
| 180815061 |  | Biswas 15 | IF | 155 | 7097 | 11925 | 1612 | 12876 | 561 | 5.26 | 37.25 | 40.85 | 101.70 | 13.58 | 18.17 | 0.50 | 52.09 | 37.90 | 151.02 | 15.26 | 15879 | 47.41 | ND* |
| 180815106 |  | Biswas 43 | IF | 124 | 5956 | 10139 | 1286 | 12456 | 525 | 4.99 | 10.66 | 39.05 | 92.54 | 10.67 | 15.87 | 0.43 | 47.57 | 30.69 | 152.20 | 11.10 | 13379 | 36.70 | ND* |
| 180815112 |  | Biswas 49 | IF | 168 | 6723 | 10667 | 1393 | 13014 | 546 | 5.22 | 11.23 | 39.07 | 100.21 | 11.96 | 16.61 | 0.51 | 53.09 | 36.76 | 119.00 | 15.16 | 14284 | 27.51 | ND* |
| 180815113 |  | Biswas 50 | IF | 146 | 6228 | 10387 | 1330 | 12289 | 562 | 5.37 | 10.96 | 37.65 | 99.12 | 11.98 | 16.58 | 0.51 | 53.80 | 35.84 | 108.67 | 13.96 | 13894 | 44.36 | 0.72 |
|  |  |  |  |  |  |  |  |  |  |  |  |  |  |  |  |  |  |  |  |  |  |  |  |
| 180815069 |  | Biswas 20 | JM | 191 | 26714 | 8563 | 1259 | 69482 | 476 | 4.15 | 12.50 | 22.27 | 119.68 | 10.74 | 63.03 | 0.54 | 43.95 | 31.15 | 24.41 | 15.47 | 12071 | 6.12 | 0.60 |
| 180815107 |  | Biswas 44 | JM | 182 | 26026 | 8055 | 1048 | 64435 | 463 | 4.29 | 10.03 | 19.10 | 121.08 | 8.94 | 54.83 | 0.48 | 39.93 | 29.59 | 22.38 | 11.23 | 10915 | 2.50 | ND* |
|  |  |  |  |  |  |  |  |  |  |  |  |  |  |  |  |  |  |  |  |  |  |  |  |
| 180815074 |  | Biswas 25 | OW | 418 | 8269 | 10661 | 2858 | 26414 | 560 | 5.19 | 13.09 | 26.88 | 116.55 | 13.61 | 39.49 | 0.45 | 63.42 | 34.29 | 17.83 | 15.47 | 15036 | 3.33 | 0.60 |
| 180815108 |  | Biswas 45 | OW | 433 | 7169 | 9703 | 2677 | 27513 | 559 | 5.01 | 11.62 | 28.58 | 116.91 | 11.43 | 38.85 | 0.45 | 61.28 | 28.19 | 14.31 | 17.32 | 12985 | ND* | 0.75 |
|  |  |  |  |  |  |  |  |  |  |  |  |  |  |  |  |  |  |  |  |  |  |  |  |
| 180815090 |  | Biswas 30 | PP | 161 | 3273 | 10932 | 1096 | 6541 | 790 | 6.85 | 11.60 | 20.19 | 100.49 | 13.13 | 18.96 | 0.48 | 67.09 | 32.15 | 22.97 | 14.95 | 13485 | 1.33 | 1.24 |
| 180815109 |  | Biswas 46 | PP | 130 | 3447 | 9856 | 866 | 9313 | 696 | 5.10 | 10.98 | 14.28 | 95.89 | 11.77 | 23.62 | 0.43 | 62.52 | 29.10 | 17.27 | 17.64 | 12730 | ND* | 0.61 |
|  |  |  |  |  |  |  |  |  |  |  |  |  |  |  |  |  |  |  |  |  |  |  |  |
| 180815095 |  | Biswas 35 | SA | 156 | 4492 | 11453 | 1119 | 12095 | 749 | 5.75 | 13.77 | 22.12 | 129.41 | 12.91 | 20.14 | 0.69 | 82.08 | 35.12 | 61.50 | 17.05 | 16056 | 2.62 | 3.00 |
| 180815110 |  | Biswas 47 | SA | 122 | 5481 | 10197 | 888 | 13721 | 681 | 5.23 | 11.56 | 20.60 | 123.95 | 11.89 | 21.95 | 0.61 | 79.25 | 35.32 | 40.42 | 14.44 | 15042 | 2.60 | ND* |
|  |  |  |  |  |  |  |  |  |  |  |  |  |  |  |  |  |  |  |  |  |  |  |  |
| 180815100 |  | Biswas 40 | WW | 171 | 14119 | 7711 | 1310 | 59596 | 448 | 3.78 | 9.79 | 19.31 | 99.57 | 8.82 | 74.57 | 0.38 | 40.48 | 23.08 | 14.47 | 12.11 | 9643 | ND* | ND* |
| 180815111 |  | Biswas 48 | WW | 248 | 17455 | 7845 | 1131 | 67203 | 474 | 4.48 | 11.82 | 19.26 | 149.30 | 8.01 | 80.52 | 0.46 | 39.50 | 23.88 | 13.64 | 10.13 | 11427 | 3.01 | ND* |

*ND: non-detectable
